# The Molecular Mechanism of Cold-Stress Tolerance: Cold Responsive Genes and Their Mechanisms in Rice (*Oryza sativa* L.)

**DOI:** 10.3390/biology13060442

**Published:** 2024-06-17

**Authors:** Nida Shahzad, Hafiz Ghulam Nabi, Lei Qiao, Wenqiang Li

**Affiliations:** 1 State Key Laboratory for Crop Stress Resistance and High-Efficiency Production, College of Life Sciences, Northwest A&F University, Xianyang 712100, China; botanist@nwafu.edu.cn (N.S.); leiqiao@nwafu.edu.cn (L.Q.); 2 State Key Laboratory of Agrobiotechnology/Beijing Key Laboratory of Crop Genetic Improvement, College of Agronomy and Biotechnology, China Agricultural University, Beijing 100193, China; hafizghulamnabi49@gmail.com

**Keywords:** rice, low temperature, cold stress and tolerance, growth and development, genes and QTLs, molecular mechanism, genetic improvements

## Abstract

**Simple Summary:**

Although the geographic distribution and production of rice are significantly influenced by temperature fluctuations, rice plants have developed a wide range of physiological, biochemical, and molecular responses to cope with and adapt to temperature stresses, including cold stress resulting from low temperatures. Deciphering cold-responsive genes and the underlying mechanisms may accelerate the development of new cold-resistant rice varieties and ensure stable rice production in adverse temperature conditions. This review aims to provide a straightforward summary of the previous progress in understanding the functions and molecular mechanisms of cold-responsive genes in rice.

**Abstract:**

Rice (*Oryza sativa* L.) production is highly susceptible to temperature fluctuations, which can significantly reduce plant growth and development at different developmental stages, resulting in a dramatic loss of grain yield. Over the past century, substantial efforts have been undertaken to investigate the physiological, biochemical, and molecular mechanisms of cold stress tolerance in rice. This review aims to provide a comprehensive overview of the recent developments and trends in this field. We summarized the previous advancements and methodologies used for identifying cold-responsive genes and the molecular mechanisms of cold tolerance in rice. Integration of new technologies has significantly improved studies in this era, facilitating the identification of essential genes, QTLs, and molecular modules in rice. These findings have accelerated the molecular breeding of cold-resistant rice varieties. In addition, functional genomics, including the investigation of natural variations in alleles and artificially developed mutants, is emerging as an exciting new approach to investigating cold tolerance. Looking ahead, it is imperative for scientists to evaluate the collective impacts of these novel genes to develop rice cultivars resilient to global climate change.

## 1. Introduction

Rice (*Oryza sativa* L.) is an important nutritious crop for more than half of the world’s population. As a temperature-sensitive crop, rice production is highly susceptible to extreme weather. However, susceptibility to environmental challenges, specifically cold stress caused by low temperatures, poses a serious threat to rice cultivation [1]. Cold stress (also called chilling stress) affects rice cultivation in several countries, including Japan, North Korea, and China. In the temperate zone, cold stress may cause significant impacts on rice production, affecting a substantial number of paddy fields. Previous research indicates that cold damage can lead to yield losses of between 0.810 and 2.740 tons/hectare, resulting in a potential decrease in grain production of up to 38.6% [2]. Moreover, when rice plants are cultivated in a greenhouse, the consecutive cold nights followed by warm days can also considerably harm plants, resulting in an 82% decrease in grain yield [3]. It is believed that subtropical and tropical plants are susceptible to cold stress and lack cold adaptation capacity. However, temperate plants usually show cold acclimation and survival in cold conditions [4]. Temperate plants are resistant to temperature fluctuations and are capable of enduring cold stress in the early spring and winter. Plants in semi-arid regions typically show vulnerability to temperature fluctuations, such as cold nights and hot days; this temperature volatility poses an extra threat to the plants [5]. It is revealed that the relative growth rates of *Artemesia ordosica* and *Artimesia sphaerosephala* are reduced at higher temperatures, specifically at 17.5/27.5 and 20/30 °C [6]. Due to global temperature fluctuations, there is an increase in extreme weather, such as extremely low temperatures, resulting in cold damage to plants, significantly reducing plant growth and yield [7].

In fact, rice plants have developed a wide range of physiological, biochemical, and molecular responses to adapt to temperature fluctuations. The responses to low temperatures may involve intricate mechanisms at different developmental stages of rice plants. Cold stress in rice mostly affects the seedling stage, which is critical for the early development and growth of rice seedlings. Cold stress affects several agronomic traits, such as shoot length, root length, and tiller number, and ultimately impacts the plant’s overall cold tolerance ability. According to previous studies, evaluation of cold-stress tolerance at the early stages of a plant’s growth, particularly at the seedling stage, is essential to identifying cold-tolerant cultivars and assessing their potential to tolerate cold stress [8].

Plant cells initially sense cold-stress signals via rigidification of the plasma membrane, Ca^2+^ influx channels (cold sensors), and receptors linked to proteins that bind with the plasma membrane. Calcium-binding proteins have the ability to detect alterations in the level of Ca^2+^ in the cytosol. These proteins generally interact with their targeted proteins, facilitate the transmission of calcium signals, and then regulate the activation of transcription factors and COR genes (cold-regulated genes). Some calcium-binding proteins also initiate the phosphorylation of the targeted proteins and coordinate the signal transduction of cold stress [9]. Furthermore, during responses to cold stress, reactive oxygen species can play a crucial role and be involved in regulating cold tolerance [10]. Elevated levels of ROS induce cellular harm, while a reduction in ROS during the initial phases of stress acts as a signal that triggers diverse stress responses. Hence, it is imperative to closely monitor the level of ROS during cold stress conditions [4,11]. Some investigations even suggest that poor chilling tolerance may lead to more accumulation of ROS as compared to high chilling tolerance, indicating that homeostasis of ROS plays a very crucial role in the regulation of cold stress tolerance in rice [12]. Moreover, it is believed that membrane fluidity may play a pivotal role in a plant’s cold tolerance. Chito oligosaccharide (COS), proline (Pro), and glutamate (Glu) amino acids also play a very significant role in rice cold resistance [13,14,15,16]. These indicate that rice responses and/or tolerance to cold stress may involve multiple and complex physiological, biochemical, and molecular mechanisms. The objective of this review is to consolidate existing knowledge and provide a comprehensive overview of the molecular mechanisms underlying cold-stress tolerance in rice.

## 2. Physiological Responses to Cold Stress

Cold stress not only causes external harm to rice, such as retardation of growth, slowing down the germination rate, lowering seed setting rate, growth inhibition, and even death of the seedlings, but also leads to metabolic and physiological alterations, including electrolyte leakage, decreases in chlorophyll content and photosynthesis rate, elevated levels of malondialdehyde (MDA), lipid peroxides, proline, sucrose, ROS, and some other metabolites [17]. It is considered that the concentrations of some plant hormones, such as gibberellin (GA), jasmonic acid (JA), and abscisic acid (ABA), are important indicators for determining cold tolerance in rice [18]. JAs and ethylene may differentially regulate the gene expression of the C-repeat binding factor (CBF) pathway [19].

Rice plants subjected to cold stress exhibit an increased uptake of soluble sugars, such as sucrose and glucose. These sugars function as osmoprotectants, aiding in the retention of turgor pressure within the cells. This response helps to mitigate the detrimental impact of cold stress on rice growth and development. Previous research has reported that cold-tolerant rice verities possess a greater abundance of genes associated with sugar metabolism pathways. This highlights the importance of sugar metabolism in enhancing cold tolerance [8,20]. Comparative transcriptome analyses have also emphasized the influence of cold stress on the metabolic pathways of soluble sugars, underscoring their vital role in rice responses to low temperatures [21]. The upregulation of genes associated with sugar metabolism highlights the plant’s ability to respond to cold stress by controlling osmotic balance and protecting its cells [22].

Cold stress in rice plants may lead to the activation of antioxidant mechanisms to fight the oxidative injury caused by reactive oxygen species (ROS). Researchers have indicated that rice plants have sustained and consistent regulation of antioxidant enzymes such as superoxide dismutase (SOD) and peroxidase (POD) when exposed to low temperatures [1]. Furthermore, the application of brassinolide (BR) to cold-stressed plants may significantly enhance the activity of antioxidant enzymes, e.g., SOD, POD, and catalase. As a consequence, there is a reduction in oxidative damage and an increase in the cold tolerance of rice seedlings [10].

The modulation of gene expression in response to cold stress in rice plants mainly involves the genes associated with stress response, metabolism, and signal transduction pathways, especially plant hormones metabolism and signal transduction [23]. When exposed to cold stress, the level of ABA in plants is observed to increase significantly, which subsequently enhances endogenous defense mechanisms. The increase in ABA levels has been found to be associated with the enhanced ability of rice cultivars to withstand low temperatures. Moreover, the regulation of genes related to the ABA signaling pathway, such as *OsABF1*, a gene encoding bZIP transcription factor, is enhanced in response to cold stress, indicating an important role of ABA in the rice response to cold stress [24,25]. Previous research also demonstrated that cold stress affects GA biosynthesis in rice plants at different developmental stages. It is revealed that *OsMKKK70* has a negative impact on rice cold tolerance by regulating GA levels in anthers during the booting stage [26]. Additionally, the germination of rice seeds is delayed by cold stress due to a reduction in GA levels by the activation of GA-catabolic genes and *SLR1*, a gene encoding the DELLA protein [27]. Cold stress triggers the accumulation of JA in rice plants, which stimulates defense mechanisms against cold-induced injury and regulates cold-responsive gene expression [28]. Furthermore, JA plays a vibrant role in plant defense mechanisms against cold stress by regulating the production of vegetation-altering compounds and volatile substances through the interaction of phytohormones [19]. JA-dependent signaling pathways are critical for improving cold tolerance and providing defense against fungal infections. In rice plants, the activation of JA signaling in response to low-temperature stress not only activates defensive structures but also affects the expression patterns of genes that are significant for cold stress tolerance and the survival of plants [29].

The mechanism of cold acclimation in plants encompasses a gradual exposure to low temperatures above the freezing point, leading to cellular and molecular alterations [30]. *Ornithine δ-aminotransferase* (*OsOAT*) has been recognized as a significant factor in enhancing cold tolerance in rice during reproductive and vegetative growth development [18]. A comparative transcriptome study revealed that both mutations of *OsOAT* and exposure to cold stress may result in comparable alterations in the patterns of gene expression within anthers [31]. Furthermore, the presence of the *OsOAT* allele in different rice cultivars suggests that specific *OsOAT* haplotypes were chosen during the domestication and breeding of cold-tolerant varieties [32]. Previous studies also demonstrated that the particular domain, i.e., the loop containing a predicted GTPase-activating protein domain, is involved in physiological responses to cold stress. It also impacts GTPase promotion as well as Ca^2+^ signaling and electrophysiological response, consistent with *CHILLING TOLERANCE DIVERGENCE 1* (*COLD1*), a biochemical function linked with G-protein signaling. It is also revealed that *COLD1* regulates G-protein signaling to enhance cold tolerance in rice, and a single nucleotide polymorphism (SNP) in *COLD1* triggers the adaptation of *japonica* rice in the chilling environment [33].

**Figure 1:**
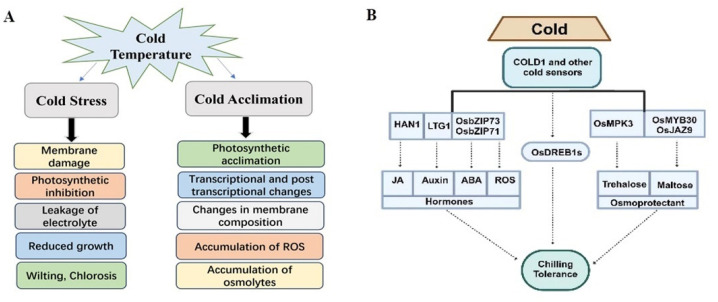
Physiological responses during cold stress. It shows a detailed recognition of the paradox in plant responses to chilling stress. (**A**) Pathway shows the sensibility of plants during chilling stress. It initiates membrane damage that minimizes photosynthetic movement and subsequent electrolyte leakage, which ultimately leads to reduced growth and plant death. (**B**) This route illustrates the complex physiological alterations, including photosynthetic acclimation, changes in membrane structure, and accumulation of ROS and osmolytes in cold-tolerant plants, demonstrating their capacity to withstand low temperatures, and the side flow diagram shows the response of various genes during cold.

Rice has developed a mechanism to detect any fluctuation in temperature, which is known as a cold-responsive pathway in which C-repeat binding factors (CBF) play a very important role. As transcription factors (TFs), CBF activates and regulates cold-responsive genes that are involved in detoxification, protection against oxidative damage, and osmotic adjustment [34]. Rice cells first detect cold signals on their cell membrane. Consequently, an important indicator of low-temperature tolerance in plants is the electrolyte leakage rate because the low temperature can change the chemical and physical properties of the membrane, which can cause fine intracellular leakage of electrolytes, as shown in Figure 1. For example, the overexpression of *OVP1*, encoding a vacuolar H+-translocating inorganic pyrophosphatase (V-PPase), leads to improved cold tolerance due to decreased electrolyte leakage in transgenic rice plants [35]. It is important to understand the physiological responses of rice plants to cold stress to develop strategies for enhancing cold tolerance in rice. By elucidating the metabolic variations and hormone signaling networks, researchers can identify the potential targets for genetic improvement and create robust rice cultivars that are capable of enduring cold stress.

## 3. Activation and Mechanism of Cold-Responsive TFs and QTLs

There are 1611 transcription factors (TFs) genes in the rice genome, and these TFs are organized into 37 families, such as the WRKY, MADS, RING finger, bZIP, MYB, NAC, bHLH, GRF, C2H2, GRAS, and TCP families [35]. The research findings from 2005 to 2024 have significantly promoted the elucidation of molecular mechanisms underlying cold tolerance in rice. These studies have eased the identification of several families of TFs that are responsible for cold responses and/or tolerance in rice at different developmental stages [36]. These TFs participate in various processes, from cold signal perception to the regulation of the expression of genes involved in cold stress.

Several signaling pathways, including Ca^2+^ signaling controlled by TFs, play an important role in regulating cold stress tolerance in rice. At the seedling stage, the protein COLD1 acts as a chilling sensor by interacting with G-protein α-subunit 1 (RGA1) to enable Ca^2+^ influx in response to cold stress, which in turn regulates the expression of the AP2/ERF transcription factor *CBF/DREB1s* [33]. It is revealed that OsMADS57, together with OsTB1, coordinates transcription of their targets, OsWRKY94 and D14, to switch its organogenesis to defense for cold adaptation in rice [37]. When exposed to cold stress, the bZIP TFs OsbZIP73 and OsbZIP71 combine to form a heterodimer, and their co-expression boosts the grain filling and seed setting rates. Moreover, the OsbZIP71/73 heterodimer not only minimizes the ABA content in anthers but also increases the transfer of sugar from anthers to pollens and enhances seed setting rate, pollen fertility, and rice grain production [38]. Furthermore, it is suggested that the early selection of OsbZIP73 facilitated the adaptation of japonica rice to cold climates [39]. In addition, the bZIP transcription factor *OsbZIP52* is strongly induced by low temperature, while overexpression of *OsbZIP52* may significantly increase sensitivity to cold stress, indicating that OsbZIP52/RISBZ5 functions as a negative regulator of the cold stress response in rice [40]. More recently, it has been revealed that histone deacetylase OsHDA716 represses cold tolerance by deacetylating bZIP transcription factor OsbZIP46 to reduce its transactivation function and protein stability [41]. OsbZIP46 regulates low-temperature-induced Ca^2+^ influx and cytoplasmic Ca^2+^ elevation through transcriptional activation of OsDREB1A and COLD1, conferring rice plants with cold tolerance. OsHDA716 deacetylating OsbZIP46 leads to the inhibition of cold tolerance in rice by preventing OsbZIP46 from binding to the target promoter, reducing protein stability and transcriptional regulation of OsDREB1A and COLD1 [41]. These investigations indicated that some TFs are at the core of the cold stress response and play critical roles in regulating rice cold tolerance.

Several NAC transcription factors have been found to play an important role in regulating cold tolerance in rice. For example, under low-temperature stress, the *OsNAC45* overexpression lines exhibited maintenance of root structure and normal growth of the seedling root, but the RNAi lines of *OsNAC45* were more sensitive to the stress [42]. Under cold-stress conditions, the NAC transcription factor *OsNAC050* was significantly up-regulated, and knocking out *OsNAC050* can increase cold-stress tolerance in rice by regulating photosynthesis and the sucrose metabolic pathway [20]. However, it is revealed that the rice NAC transcription factor *ONAC095* plays opposite roles in drought and cold stress tolerance [43]. A recent investigation has shown that the bHLH transcription factor OsbHLH57 can enhance chilling tolerance in rice at diverse developmental stages [44]. Overexpression of *bHLH57* enhanced cold tolerance by increasing trehalose synthesis, whereas the loss-of-function mutants of *bHLH57* are more sensitive to cold stress and have reduced trehalose in rice. Furthermore, it is revealed that bHLH57 may regulate ROS metabolism and CBF/DREB-dependent pathways in response to cold stress [44].

Interestingly, it is shown that overexpression of the tomato AP2/ERF transcriptional factor *TERF2* enhances cold tolerance in transgenic rice without altering its growth or agronomic traits [45]. Physiological assays revealed that *TERF2* promotes the accumulation of chlorophyll and osmotic substances while reducing the levels of MDA, ROS, and electrolyte leakage in rice plants under chilling stress. On molecular levels, *TERF2* activates the expression of cold-responsive genes, including *OsICE1*, *OsSODB*, *OsFER1*, *OsMyb*, *OsCDPK7*, *OsTrx23*, and *OsLti6*, in rice transgenic plants under natural conditions or cold stress, indicating a potential utility of TERF2 in improving rice cold tolerance [45]. Recently, a comprehensive investigation of the AP2/ERF transcription factor OsERF096 reveals the multiple regulatory roles of *OsERF096* in cold stress responses, probably by regulating sucrose metabolism and auxin IAA accumulation and signaling pathways [46]. These studies indicate that TFs may activate the cold-stress response and/or tolerance in rice by regulating different target genes.

So far, most of the cold-resistant genes of great importance in rice breeding or production have been identified as QTL (quantitative trait loci). To identify QTLs for low-temperature resistance in rice, a group of recombinant inbred lines (RIL’s) was created by using rice indica cultivar 93–11 and japonica cultivar Nipponbare and then used for QTL mapping; subsequently, a total of five quantitative trait loci (QTL’s) were discovered that are responsible for the cold tolerance in rice at the seedling stage [33]. These great efforts led to the discovery of the most important QTL for cold tolerance, the *COLD1*, a cold sensor in rice [33,47]. It was indicated that one SNP in COLD1 determines cold tolerance during rice domestication [48]. An integrated global analysis further revealed a vitamin E-vitamin K1 sub-network downstream of COLD1, conferring cold tolerance divergence in rice [49].

Previously, a total of seven cold-tolerant QTLs were identified in rice by using F_2_ and F_3_ populations through a controlled breeding process involving the cold-tolerant cultivar IL112 and the cold-sensitive cultivar Guichao2. Subsequently, microarray-assisted fine-mapping of the cold-tolerant QTLs leads to the identification of *LTT7*, as overexpression of this gene also increases cold tolerance in rice seedlings [50]. It was probable that *LTT7* enhanced cold tolerance in rice by mediating the DREB/CBF pathway [50]. *Cold tolerance at booting stage-1* (*Ctb1*), a QTL associated with chilling tolerance during the booting stage, was first discovered and mapped inside a genomic segment of 56 kilobases [51]. Subsequent investigations have further fine-mapped the QTL’s location to a smaller region of 17 kilobases [52]. Another QTL for cold tolerance, *CTB4a* (*cold tolerance at the booting stage*), encodes a conserved leucine-rich repeat receptor-like kinase [53]. It was shown that different *CTB4a* alleles confer distinct levels of cold tolerance, and selection for variation in the CTB4a promoter region has occurred on the basis of environmental temperature [53]. Furthermore, CTB4a can interact with AtpB (ATP synthase beta subunit) under cold-stress conditions, and upregulation of *CTB4a* correlates with ATP content by enhancing the activity of AtpB [53].

## 4. Signal Transduction and Membrane Stability

It has long been accepted that the transient elevation of cytoplasmic calcium acts as a critical signal for plant cold tolerance. Previous research has demonstrated that Ca^2+^ signaling controls the activation and transmission of cold signals [54]. Ca^2+^ could serve as the second messenger molecule produced just after the cell detects a drop in temperature from the environment. The increase in cytoplasmic Ca^2+^ concentrations leads to the activation of cold-responsive genes such as *OsCDPK-24*, CBL interacting protein kinase 7, and multi-stress responsive gene 2, which are located farther downstream [55,56,57]. At the seedling stage, the cold sensor COLD1 interacts with RGA1 to enable Ca^2+^ influx in response to cold stress, which in turn regulates the expression of the AP2/ERF transcription factor *CBF/DREB1s* [33]. Moreover, as described previously, OsbZIP46 regulates cold-stress-induced Ca^2+^ influx and cytoplasmic Ca^2+^ elevation through transcriptional activation of OsDREB1A and COLD1, conferring rice plants with cold tolerance. OsHDA716 deacetylates OsbZIP46, which prevents OsbZIP46 from binding to the target promoter, including reducing the expression of *OsDREB1A* and *COLD1* [41].

According to a recent study, the rice *OsCNGC9* encoding cyclic nucleotide-gated ion channel positively regulates cold tolerance by mediating cold-induced calcium influx and cytoplasmic calcium elevation [58]. The overexpression of *OsCNGC9* increases cold tolerance, whereas the *OsCNGC9* dysfunctional mutant is more sensitive to continuous low-temperature environments and lacks calcium influx [58]. Furthermore, it demonstrates that, in response to cold stress, OsSAPK8, a homolog of Arabidopsis thaliana OST1, phosphorylates and activates OsCNGC9 to trigger Ca^2+^ influx; the transcription of OsCNGC9 is activated by the transcription factor OsDREB1A [58]. In addition, it is indicated that OsCNGC14 and OsCNGC16 are also required for cold tolerance and are modulators of calcium signals in response to temperature stress because the deletion of *OsCNGC14* and *OsCNGC16* diminished and eliminated Ca^2+^ signals triggered by cold stress [59]. A recent study identifies a novel cold-sensing mechanism that simultaneously conveys cold-induced protein conformational change and enhances kinase activity and Ca2+ signal generation to facilitate chilling tolerance in rice [57]. Previously, it was indicated that a point mutation in CBL-interacting protein kinase 7 (OsCIPK7) led to a conformational change in the activation loop of the kinase domain, subsequently leading to an increase in protein kinase activity, thus conferring an increased tolerance to cold stress [56]. In a recent investigation, it was reported that Calreticulin 3 (OsCRT3) localized at the endoplasmic reticulum (ER) exhibits conformational changes under cold stress, thereby enhancing its interaction with OsCIPK7 to sense cold [57]. OsCRT3 localizes at the ER and mediates increases in cytosolic calcium levels under cold stress; however, cold stress triggers secondary structural changes in OsCRT3 and enhances its binding affinity with OsCIPK7, which finally boosts its kinase activity [57].

The fluidity of the phospholipid membrane in the cell can alter in response to low temperatures, which in turn is crucial for the plant’s response to cold stress [60]. The perception of cold stress signals in plant cells is a complex process that involves the complicated interplay of several factors within the plasma membrane [61,62]. Cold stress triggers a tightly regulated mechanism that increases the level of unsaturated fatty acids in cells [61]. This adjustment ensures the appropriate fluidity of the cell membrane, facilitating growth in low-temperature conditions [60,61]. For example, the gene *OVP1*, which encodes inorganic pyrophosphatase that translocates H^+^ into the vacuole (VPPase), confers chilling tolerance by lowering the concentration of MDA and increasing the amount of proline, which concurrently improves the integrity of the cell membrane [35,63]. As evidenced by a decrease in the number of fatty acids and a reduction in the fluidity of the membrane in *OsFAD8* knockout mutants, the gene *OsFAD8* is essential for the adaptation of rice to cold stress [64].

Alterations in membrane stiffness, the osmotic pressure, and the physical state of proteins present on the membrane are the three factors that allow plant cells to sense the effect of cold stress [65]. Low temperatures cause a change in membrane rigidity, resulting in increased electrolyte leakage, as an indicator of the activation of cold-tolerant genes such as *OsNAC5*, *TERF2*, and *OVP1* [45,66]. When membrane stiffness and ion conductance increase, a signal cascade of cold-activated MAPK will be triggered within the cells [15]. In addition, calcium channels can be activated due to low-temperature stress-induced membrane rigidification, which promotes the entry of Ca^2+^ into the cytoplasm as a starting event in chilling stress [67]. These cold-sensing signals are capable of being amplified and interpreted by a cascade of calcium signals, ultimately activating the DREB CRT/DRE pathway, which is essential for perceiving and responding to cold stress in rice [61].

**Figure 2:**
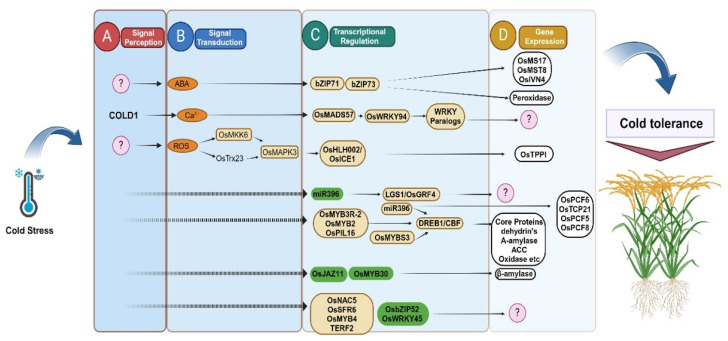
Flow chart diagram of signal transduction and membrane stability. Presents the coordinated molecular response of rice plants during cold stress. (**A**) Cascade is started by an immediate signal perception. (**B**) Signal transduction pathways quickly come into action, opening the door for (**C**) the participation of several transcriptional regulatory elements, and (**D**) the plant’s defense mechanism against cold stress is the result of the subsequent gene expression.

Although significant progress has been made in the elucidation of the mechanism of cold-stress signal transduction in rice, there are still many unclear aspects about the gene regulatory networks, and it will be challenging to determine the mechanisms involved in the chilling stress tolerance of rice. Figure 2 represents the detailed molecular mechanisms of signal transduction and membrane stability in rice responses to cold stress.

## 5. Genes Identified in Rice during Cold Stress at Various Developmental Stages

In the past few decades, the significant progress made in molecular biology has significantly enhanced our understanding of how plants respond to cold stress at various levels [61,67]. As a model plant and an important food crop, numerous genes associated with cold responses have been identified by various molecular technologies (Table S1). These genes are involved in various aspects of rice growth, development, and environmental adaptation to cold stress. Researchers have gained important insights into the molecular pathways that enable rice plants to adapt and survive in cold climates by examining their mechanisms. As indicated in Table 1, some of these genes have been successfully cloned and have provided novel possibilities for both theoretical and practical investigation.

It is not only exciting from a scientific point of view to understand the molecular mechanisms underlying plants' response to cold stress, but it also has enormous potential to improve agriculture. Scientists can contribute to the development of improved crop cultivars that are more tolerant to low temperatures by locating and analyzing these genes. Thus, even in regions susceptible to cold weather, this can ensure a consistent global agricultural yield. Studying these genes has the potential to significantly improve plant breeding efforts, providing a notable advantage. Through the use of these genes in breeding processes, scientists can develop new crop varieties that are more resilient to adverse climate conditions.

## 6. Application of Omics Technologies in the Identification of Cold-Stress Response Genes or Pathways

The integration of several omics’ datasets, such as ionomics, metabolomics, transcriptomics, and proteomics, is an effective tool for comprehensively exploring the essential metabolites, genes, proteins, and networks associated with cold-stress responses and tolerance [83,84]. Among these omics technologies, transcriptomic methodologies are most widely used to investigate cold-stress responses and tolerance in rice plants [8,21,105]. A transcriptomic dynamic study revealed that two rice *indica* cultivars, namely SQSL and xzx45, exhibit different levels of tolerance to low temperatures due to differential expression of cold-responsive genes [8]. Another group has performed a comparative transcriptomic study in rice, which discovered the differentially expressed genes (DEGs) linked to the metabolism of lipids, carbohydrates, and proteins during cold stress [21]. Transcriptomics profiling was also performed on cultivated rice and weedy rice in response to cold stress [105]. Many common and special DEGs were identified in cold-tolerant and cold-sensitive genotypes of cultivated rice and weedy rice, respectively [105]. Several cold stress-responsive genes, including the leucine-rich repeat domain (LRR) gene and the basic helix loop helix (BHLH) gene, were found in cold-tolerant varieties as compared to cold-sensitive verities. The gene ontology (GO) enrichment analysis further enhanced our understanding of biological processes, molecular functions, and cellular components associated with cold-responsive pathways [105].

In addition, to identify key proteins and elucidate their underlying mechanisms that control cold tolerance in rice, some investigations also performed comparative proteomic studies on rice varieties with different cold tolerances. It was reported that the combination of 2D electrophoresis and mass spectrometry (MS) techniques was used to investigate the difference in cold tolerance between two distinct rice cultivars [106]. Through their analysis, a total of 59 proteins were associated with the observed differences in cold tolerance [106].

Recent studies have effectively used quantitative proteomic methodologies such as isobaric tags for comparative and absolute quantification (iTRAQ) and tandem mass tags (TMT) to analyze the changes in the proteome in response to cold stress in rice [107]. These proteomic technologies exhibit notable efficacy and consistency as well as high throughput, making them valuable tools for studying cold-stress responses in rice. Currently, due to cost reduction and technological accessibility, integrated multi-omics analyses have been a mainstream tool to investigate the molecular mechanisms underlying cold-stress tolerance in rice [84].

## 7. Rice Breeding for Cold Tolerance

The main objective of breeding efforts over the ages has been to increase grain output, which has remained the ultimate goal since the beginning of modern rice breeding in the early 19th century [108]. Based on the discovery of numerous cold-tolerance-associated genes, or QTLs, such as *COLD1*, genomic breeding has become more important for the development of cold-tolerant cultivars in rice [48]. For example, it has been found that the native *CTB4a* alleles provide varying levels of resistance to low temperatures at the booting stage, while the natural variation taking place in the promoter area of *CTB4a* during domestication conferred cold tolerance. So, artificial selection is an effective strategy for maintaining a haplotype with improved resistance to cold stress [53].

The application of genomic breeding to enhance cold tolerance or other critical agronomic properties has grown significantly. For example, the excellent rice *japonica* cultivar Kongyu 131 is commonly grown in northern China and has the advantages of early maturity, cold tolerance, superior quality, broad adaptability, high yield, etc. However, it is still important to increase the yield of Kongyu131 by introducing a high-yielding allele, such as the introduction of the *Gn1a* (*GRAIN NUMBER1a*) gene [109]. In this respect, the genome of Kongyu 131 has been modified by the transfer of a tiny chromosomal segment (~800 kb) from the rice indica variety to enhance its ecological adaptability and allow its cultivation in regions of low latitude [110]. An effective breeding program focused on the development of rice varieties with cold tolerance has involved the assessment of multiple rice accessions for their ability to tolerate cold at various developmental stages [111]. By employing genotypic assays targeting specific markers such as COLD1 and NAC6, researchers were able to identify accessions with strong cold tolerance and find new markers that accurately predict cold tolerance. The development of new varieties has made a substantial contribution to improving rice cold-stress tolerance, resulting in increased yields in cold climates [112].

In rice breeding, marker-assisted selection (MAS) is a tool primarily used for three main purposes: (1) gathering desirable alleles by following their inheritance patterns through generations as dominant or recessive genes; (2) locating desirable individuals in a separated breeding population; and (3) integrating important alleles. In this respect, marker-assisted backcrossing (MABC) has been the most efficient and popular way to integrate desirable alleles [113,114]. MABC, involving transferring a specific region from one parent to another, can lead to significant enhancement of characters when a gene exerts important impacts on a significant number of observable traits or when the appearance of a desired trait is controlled by a single gene. Rice breeders prefer this method due to its cost-effectiveness as compared to other methods [115]. Therefore, the development of cold resistance in rice is mainly possible through (a) marker-assisted backcross, (b) pedigree selection based on marker, and (c) using conventional breeding practices.

## 8. Novel Rice Management Strategies to Induce Cold Tolerance

Improving the ability of rice to withstand low temperatures is crucial for maintaining high levels of rice production in the face of climate change and ensuring an adequate food supply for a growing population. Novel techniques such as genetic methodologies, microbiological manipulations, and seed treatment with external stimulants provide exciting opportunities to enhance the ability of rice to withstand cold stress. Genetic techniques and microbial manipulations have been proven to be effective strategies for improving cold tolerance in rice. Key genes, such as *LTT1* [96] and *OsbZIP54* [116], could serve as possible targets for molecular breeding. By utilizing genetic transformation, scientists can accelerate the production of rice cultivars resistant to cold stress.

Utilizing microbial interventions is a viable method to improve the ability of rice to withstand cold temperatures. Specific rhizospheric bacteria, including *Staphylococcus* sp. CSR1T2 and *Kosakonia* sp. CIR2, have demonstrated the capability to enhance the ability of rice plants to withstand cold stress through different mechanisms. These beneficial microorganisms can interact with the plant’s root system, regulating physiological processes and improving stress tolerance mechanisms, ultimately strengthening the plant’s capacity to endure cold stress [117]. Moreover, the application of external stimulants during seed priming provides an effective way to improve the ability of rice seedlings to withstand cold stress. Seedlings can be prepared to activate their defense mechanisms against cold stress during germination by treating seeds with certain compounds or substances before sowing. The more important thing is that this strategy enhances both the survival ability of seedlings in cold conditions and the overall growth and development of the plant throughout its lifespan [118].

## 9. Conclusions and Outlooks

Despite extensive research in the past two decades, the molecular mechanisms responsible for perceiving and transmitting cold signals in plants still remain unknown. Rice production is negatively affected by low temperatures, particularly in cold areas where rice is cultivated. The development of new cold-resistant rice cultivars is crucial. Integrating genetic engineering and traditional breeding can lead to successful breeding programs for developing new rice varieties.

In this review, we discussed both historical and contemporary research on rice responses to cold stress. We shed light on the role of various cold-stress-associated genes, like *COLD1*, *OsCUGT1*, *OsLTT1*, *OsbZIP54*, *OsCDP*, and *OsMYB30*, in response to cold stress. These cold-responsive genes can be introduced into freezing-sensitive rice cultivars through different breeding techniques to improve cold tolerance. This will facilitate the development of novel approaches to improve rice cold tolerance and ensure global food security in the face of the challenges resulting from climate change.

Based on the observations and findings of this review, we suggest that modifying some key genes like *COLD1*, *OsCUGT1*, *OsLPXC*, *OsCTB2*, *OsLTT1*, *OsPIN5B*, *OsGS3*, and *OsMYB30* will help researchers and breeders develop cold-tolerant rice varieties. This will eventually enhance productivity. Integrating genetic discoveries with practical field applications remains a critical challenge in establishing successful cold-tolerant rice varieties. As a result, additional investigations that are broader and more comprehensive are urgently required in this emerging field of inquiry.

## Figures and Tables

**Table 1:** Some rice genes are identified at different developmental stages for cold tolerance (Errows: ↑ Increase).

Gene	Developmental Stages	Functions of Genes	Reference
*bHLH57*	Flowering, Booting, Germination	Cold tolerance and grain yield improvement ↑	[44]
*OsPIN9*	Seedling stage	Regulation of auxin, ROS homeostasis and cold tolerance ↑	[68,69]
*NOG1*	Seedling stage	Grain number and yield during cold ↑	[70]
*OsDREB1B*	Seedling stage	Regulate chilling tolerance ↑	[71]
*OsCML16*, *OsPILS7a*	Seedling Stage	Regulate primary root elongation and cold tolerance ↑	[72]
*OsMTACP2*	Seedling stage	Mediated wax ester biosynthesis and cold tolerance ↑	[73]
*OsERF096*	Different stages	Regulation of cold stress ↑	[46]
*OsSPL7*	Maturity stage	Rice growth and stress responses ↑	[74]
*OsCUGT1*	Germination, reproductive	Rice height and spikelet fertility ↑	[75]
*OsCRT3*	Seedling stage	Regulator of chilling tolerance ↑	[57]
*COLD11*	Germination stage	Chilling tolerance ↑	[76]
*OsLPXC*	Reproductive stage	Regulate cold tolerance ↑	[77]
*OsLUX*	Seedling stage	Cold stress and circadian rhythm ↑	[78]
*OsSPXs*	Different stages	Rice adaptation to cold stress ↑	[79]
*OsNAC5*	Germination and seedling	Cold tolerance ↑	[80]
*qCTB7*	Booting stage	Regulates the appearance and morphology of the anthers and pollen ↑	[81]
*COG1*	Germination stage	Cold tolerance ↑	[82]
*OsOAT*	Germination stages	Male fertility, cold tolerance ↑	[32]
*OsHPL1*	Seedling, germination	Modulates rice metabolism ↑	[83]
*OsSEH1*	Seedling stage	Cold tolerance ↑	[84]
*OsHis1*	Germination and seedling	Tolerance to temperature stress ↑	[85]
*OsMAPK3 OsLEA9*	Reproductive stage	Cold tolerance ↑	[86]
*OsSAPK6*	Seedling stage	Activate cold resistance	[87]
*OsLsi1*	Seedling stage	Enhances microbe-plant interactions and cold tolerance ↑	[88]
*OsWRKY115*	Seedling stage	Cold tolerance ↑	[89]
*OsGATA16*	Seedling stage	Cold tolerance ↑	[90]
*OsCTB2*	Booting stage	Cold adaptation ↑	[91]
*OsCNGC9*	Seedling stage	Enhanced cold tolerance ↑	[58]
*OsPIN5b*, *GS3*, and *OsMYB30*	Reproductive stage	Increased panicle length, enlarged grain size, enhanced cold tolerance ↑	[92]
*OsETR4*	Seedling stage	Seedling survival rate ↑	[93]
*OsLsi1*	Germination stages	Enhanced the antioxidant system and non-structural carbohydrates ↑	[94]
*OsTMF*	Different stages	Regulate chilling tolerance by affecting cell wall properties	[95]
*OsLTT1*	Booting stage	Cold tolerance Maintaining tapetum degradation and pollen development ↑	[96]
*OsLTG5*	Seedling stage	Cold tolerance ↑	[97]
*OsUGT90A1*	Seedling stage	Protect the plasma membrane and promote leaf growth ↑	[98]
*OsHAN1*	Different stages	Chilling tolerance ↑	[99]
*Ghd8*	Different stages	Flowering time, heading date ↑	[100]
*OsDREB1G*	Seedling and germination	Cold stress response ↑	[101]
*OsRAN2*	Different stages	Regulate export of intranuclear tubulin and cell division ↑	[102]
*OsWRKY71*	Seedling stage	Functions as a transcriptional repressor ↑	[103]
*COLD1*	Seedling stage	Chilling tolerance ↑	[33]
*Osa-miR319b*	Different stages	wider leaf blades and delayed development ↑	[104]

## Data Availability

The data presented in this study are available in the article.

## Supplementary Materials

The following supporting information can be downloaded at: https://www.mdpi.com/article/10.3390/biology13060442/s1, Table S1. A detailed list of genes identified in rice for cold responses and/or cold tolerance.
